# Biological Control Potential of the Reduviid Predator *Rhynocoris fuscipes* (Fabricius) in Managing Noctuid Pests: Insights Into Predation and Prey Preference

**DOI:** 10.3390/insects16020224

**Published:** 2025-02-18

**Authors:** Chuanzhen Xue, Jiaying Mao, Bowen Xu, Lei Zhou, Haihang Zhou, Jianjun Mao, Zhongjian Shen, Lisheng Zhang, Mengqing Wang, Yuyan Li

**Affiliations:** 1 State Key Laboratory for Biology of Plant Diseases and Insect Pests, Key Laboratory of Natural Enemy Insects, Ministry of Agriculture and Rural Affairs, Institute of Plant Protection, Chinese Academy of Agricultural Sciences, Beijing 100193, China; xchuanzhen@163.com (C.X.); mjy20190802@163.com (J.M.); xubowen981001@163.com (B.X.); zhoulei811@126.com (L.Z.); 15938792741@163.com (H.Z.); maojianjun0615@126.com (J.M.); shenzhongjian@caas.cn (Z.S.); zhanglisheng@caas.cn (L.Z.); 2 Key Laboratory of Animal Biosafety Risk Prevention and Control (North), Ministry of Agriculture and Rural Affairs, Shanghai Veterinary Research Institute, Chinese Academy of Agricultural Sciences, Shanghai 200241, China

**Keywords:** predatory bug, functional response, predation ability, intraspecific interference competition, prey preference

## Abstract

Understanding predator–prey and predator–predator interactions is essential for evaluating the effectiveness of biocontrol agents and developing efficient pest management strategies. This study investigates the effects of prey species, predator life stage, and predator density on the predatory efficiency of the generalist predator *Rhynocoris fuscipes* (Fabricius) under semi-field conditions. Both the nymphs and adults of *R. fuscipes* consumed significantly more second-instar larvae of *S. frugiperda* than larvae of *S. litura* or *M. separata*. Notably, fifth-instar nymphs and adults exhibited higher predation capacity (11.75 ± 0.37 and 10.90 ± 0.40 larvae) than fourth-instar nymphs (9.05 ± 0.29 larvae) on *S. frugiperda*. *R. fuscipes* demonstrated a Type II functional response at all developmental stages toward each prey species, and fifth-instar nymphs revealed higher attack rates (*a* = 1.5205 ± 0.0544) on *S. frugiperda*; additionally, the handling time did not significantly differ among three prey species. It suggests that this predator may be more effective at controlling noctuid populations at low prey densities. As predator age and density increased, intraspecific competition among *R. fuscipes* also intensified. In a multi-prey system, *R. fuscipes* showed a marked preference for *S. frugiperda* over the other two prey species. These findings indicate that the fifth-instar nymphs and adults of *R. fuscipes* are particularly effective in suppressing early-instar larvae of the three noctuid pests, especially *S. frugiperda*, at low densities. This study enhances our understanding of the predation capacity and prey preference of *R. fuscipes* towards the three noctuid pests and provides a foundation for the development of more targeted and efficient pest management strategies using this predator.

## Simple Summary

The noctuid species *Spodoptera frugiperda* (J. E. Smith), *Spodoptera litura* (Fabricius), and *Mythimna separata* (Walker) are among the most destructive agricultural pests globally. Currently, the management of these pests largely relies on chemical control methods, which are often inefficient, contribute to the development of insecticide resistance, and pose risks to environmental and food safety. In contrast, biological control using natural enemies, such as predators and parasitoids, has proven to be an effective, environmentally safe, and sustainable alternative. This approach has been successfully implemented in various regions of Africa, Asia, and the Americas. The assassin bug *Rhynocoris fuscipes* (Fabricius) is a key generalist predator that effectively controls a wide range of pests, including caterpillars, aphids, and planthoppers. However, its predation efficiency against the fall armyworm, common armyworm, and oriental armyworm remains poorly understood. In this study, we assessed the potential of *R. fuscipes* as a candidate biocontrol agent for managing these armyworm pests by examining its functional response, intraspecific interference competition, and prey preference. Our findings suggest that *R. fuscipes* shows strong potential as a biocontrol agent against these noctuid pests and provide valuable insights for optimizing its use in integrated pest management strategies.

## 1. Introduction

Armyworms are pests that attack a wide range of crops, especially cereals [1,2,3]. Most armyworm species cause severe damage by feeding gregariously and voraciously on leaves and seedlings [4]. Common armyworm species, including the fall armyworm *Spodoptera frugiperda* (J. E. Smith) (Lepidoptera: Noctuidae), the common armyworm *Spodoptera litura* (Fabricius) (Lepidoptera: Noctuidae), and the oriental armyworm *Mythimna separata* (Walker) (Lepidoptera: Noctuidae), are all highly polyphagous agricultural pests with a global distribution [5,6,7]. Due to their rapid growth, large food intake, high reproductive rate, and strong dispersal capacity, these pests have become highly destructive, causing significant economic losses in various crops [8,9,10]. The notorious migratory pest *S. frugiperda*, native to the tropical and subtropical regions of the Americas, was first detected in Central and Western Africa in early 2016. These pests caused annual maize losses of up to 17.7 million tons in 12 African countries, a loss large enough to feed tens of millions of people in 2018 [11,12,13]. Subsequently, it invaded China in December 2018, then spread through 26 provinces (autonomous regions and municipalities) in 2019 and 27 in 2020, damaging 1.125 and 1.278 million hectares of crops, respectively [14]. The common cutworm *S. litura* is widely distributed across temperate and subtropical regions, with its larvae capable of feeding on over a hundred economically important crops, including maize, rice, soybean, and cotton [15,16]. In pepper fields, large infestations of *S. litura* can damage the bare stalks, leading to significant losses [17]. The oriental armyworm *M. separata* is a migratory and polyphagous pest species found throughout Asia, Oceania, and several Pacific islands. It causes severe damage to rice, maize, wheat, vegetables, and other crops [18]. Between 2012 and 2013, an outbreak of *M. separata* in China threatened approximately 1743.7 million hectares of farmland, and this threat has continued in recent years [18]. The frequent or sudden appearances of these armyworms have led to extensive insecticide usage, resulting in increased pesticide resistance among pests and numerous adverse effects on non-target organisms [19,20,21,22]. Therefore, an integrated and sustainable pest management strategy is essential for the prevention and control of these lepidopteran pests, which is crucial for agricultural production and global food security [23,24,25].

Augmenting and conserving natural enemies to mitigate the impacts of pests is a fundamental strategy in biological control, which is both environmentally sustainable and cost-effective strategy [26]. This approach plays a crucial role in integrated pest management (IPM) programs. Among the biological control agents, Heteropteran predatory bugs are known for their broad prey range, with several species demonstrating significant predation capacity against key agricultural pests such as *S. frugiperda*, *S. litura,* and *M. separata* [27,28,29,30,31,32,33]. Notable examples include *Arma chinensis* (Fallou) (Hemiptera: Pentatomidae) [27,28,29], *Picromerus lewisi* (Scott) (Hemiptera: Pentatomidae) [30,31,32], and *Rhynocoris marginatus* (Fabricius, 1794) (Hemiptera: Reduviidae) [33]. However, only a few species of predatory bugs, such as *Podisus maculiventris* (Say) (Hemiptera: Pentatomidae), *Cantheconidea furcellata* (Wolff) (Hemiptera: Asopinae), *Geocoris spp.* (Hemiptera: Lygaeidae), and *Orius sauteri* (Poppius) (Hemiptera: Anthocoridae), have been successfully mass-reared for commercial use in field or greenhouse applications [34]. The selection and evaluation of additional potential predators are critical for advancing biological control strategies against major agricultural pests.

The assassin bug *Rhynocoris fuscipes* (Fabricius) (Heteroptera: Reduviidae) is a generalist predator that is widely distributed across East Asia, including China, India, Japan, and Vietnam [35]. Both the nymphs and adults of this predator prey on a variety of agricultural pests, such as the brown planthopper *Nilaparvata lugens* (Stal) (Hemiptera: Delphacidae), the mealybug *Phenacoccus solenopsis* (Tinsley) (Hemiptera: Pseudococcidae), the cotton stainer *Dysdercus cingulatus* (Fab.) (Hemiptera: Pyrrhocoridae), *Dysdercus koenigii* (Fab.) (Hemiptera: Pyrrhocoridae), and several aphid species [36,37,38,39]. As an effective biological control agent for agriculture pests, *R. fuscipes* is commonly found in agroecosystems throughout southern China [39]. Given its polyphagous nature, this predator may also play significant role in controlling noctuid pests. Understanding its prey range, prey preference, and predation capacity is essential for optimizing its use in biological control programs.

Functional response describes the number of prey consumed per unit time by a predator as prey density changes [40]. It is a crucial tool for evaluating the potential of biological control agents, as it quantifies key aspects of predation behavior, foraging ecology, digestion, and fitness-related factors [19,29,41,42,43]. Holling (1959) identified three types of functional response (Type I, Type II, and Type III) [44]. The most commonly observed and widely used types in predator–prey studies are Type II and Type III, where per capita predation increases curvilinearly and sigmoidally with prey density, respectively [44,45]. In contrast, Type I represents a linear relationship between prey density and the maximum number of prey killed. In previous research, functional responses are typically measured in Petri dishes under laboratory conditions [46]. In addition to prey density, the functional response can also be influenced by various factors, including predator density, competitor densities, temperature, predator age, spatial factors, host plants, and the use of insecticides [47,48,49,50,51]. To enhance the efficacy of predators in pest suppression, it is crucial to study their consumption ability through functional response assessments under field-simulated conditions. Furthermore, analyzing intraspecific competition and prey preference is vital for determining a predator’s predation capacity and evaluating its potential as a candidate for biological control programs [52,53].

In this study, we evaluated the potential of *R. fuscipes* as a biological control agent for managing three major noctuid pests (*S. frugiperda*, *S. litura,* and *M. separata*) by conducting functional response and intraspecific interference competition experiments under semi-field conditions. The functional response of the fourth- and fifth-instar nymphs, as well as adult *R. fuscipes*, to second-instar larvae of each prey species was assessed on maize plants within cages. In addition, we examined intraspecific interference competition among *R. fuscipes* at different developmental stages. This study also compared the consumption rates and prey preference of *R. fuscipes* for *S. frugiperda*, *S. litura,* and *M. separata*. The findings will offer valuable insights into the predator–prey and predator–predator interactions between *R. fuscipes* and its prey, contributing to the development of more effective strategies for controlling these noctuid pests using *R. fuscipes*.

## 2. Materials and Methods

### 2.1. Insect Colonies

The colony of the assassin bug *R. fuscipes* was originally collected from a field in Guangdong, China, and has been maintained in our laboratory in Beijing for over three generations. Both nymphs and adults of *R. fuscipes* were reared in a cage (6 cm length, 6 cm width, and 1.5 cm height) with *Tenebrio molitor* (Coleoptera: Tenebrionidae) under controlled conditions of 28 ± 1 °C, 16 h of light per day (16L:8D), and 70 ± 5% relative humidity (RH) in an artificial climate chamber. For experimental purposes, fourth- and fifth-instar nymphs and adults of *R. fuscipes*, selected 2 to 3 days after molting or eclosion, were used. These insects were starved for 24 h before being placed into experimental arenas to assess their functional response, intraspecific interference competition, and prey preference.

Colonies of *S. frugiperda*, *S. litura*, and *M. separata* have been maintained in our laboratory for at least two years. The larvae of these species were reared on fresh maize leaves and improved artificial diets as described for *S. frugiperda* [54,55], *S. litura* [55], and *M. separata* [55], respectively. Adults were provided with a 10% sucrose solution. All insect colonies were reared in climate chambers under conditions of 26 ± 1 °C, 16L:8D, and 70 ± 5% RH. Second-instar larvae (molted within 1 to 2 days) of each prey species were used in all subsequent experiments.

### 2.2. Experimental Conditions

To create experimental arenas that closely mimic natural conditions, we assessed the predation rate and prey preference of *R. fuscipes* towards armyworms on maize plants within mesh cages (100 mesh, 20 cm in length, 20 cm in width, and 35 cm in height). Each cage contained a flowerpot (15.5 cm top diameter, 11.5 cm bottom diameter, and 14 cm height) in which fifteen maize seedlings were planted, growing until they reached the two-leaf stage (after 14 days). Prey individuals were randomly distributed across all maize plants. All experiments were conducted in an artificial climate chamber at 28 ± 1 °C, with a photoperiod of 16L:8D and 70 ± 5% RH. Prey individuals were not replaced during the experiments.

### 2.3. Functional Response

To examine the functional response of *R. fuscipes* to different prey densities, we tested the consumption rates of fourth- and fifth-instar nymphs, as well as male and female adults, on armyworms at prey densities of 5, 10, 15, 20, and 30 larvae per predator (24 h starved). After 24 h, the number of armyworms consumed by the predator was recorded. Control treatments, without predators, were also used to assess the natural mortality of the prey species, enabling the correction of prey consumption rates for natural mortality. Each treatment included 20 predators as replicates at each prey density.

### 2.4. Intraspecific Interference Competition

The effects of intraspecific interference competition on the predation rate and foraging behavior of *R. fuscipes* were also investigated. This was carried out using fourth- and fifth-instar nymphs, as well as male and female adults, exposed to prey densities of 50, 100, 150, 200, and 250 armyworms, with varying numbers of *R. fuscipes* predators (1, 2, 3, 4, or 5 individuals) in the experimental arenas. The prey-to-predator ratio was maintained at 50 for each group of predators placed in a cage. As the number of predators increased, so did competition for space. After 24 h, the number of surviving prey was counted. The daily predation rate by *R. fuscipes* was calculated, and the coefficients of intraspecific interference were analyzed. Each treatment was replicated 10 times.

### 2.5. Prey Preference

To assess the predation preferences of *R. fuscipes* for different prey species, including *S. frugiperda*, *S. litura*, and *M. separata*, experiments were conducted using 24 h starved *R. fuscipes* nymphs (fourth and fifth instars) and adults (both females and males). In each trial, 10 instar larvae from each prey species (total of 30 prey larvae) and 1 predator were introduced into an experimental arena. The number of prey consumed by each stage of *R. fuscipes* was recorded, and the total predation rate for each developmental stage was calculated. For each predator life stage, 10 replicates were performed, with one predator per repetition.

### 2.6. Statistical Analyses

In the control groups, no mortality of *S. frugiperda*, *S. litura*, or *M. separata* larvae was observed, and therefore, prey mortality data were not included in the analysis. A one-way analysis of variance (ANOVA) was used to compare prey consumption and predation rates across different developmental stages of *R. fuscipes* at varying prey densities. Significant differences between treatments were identified using Duncan’s multiple range test (*p* < 0.05). Additionally, comparisons of prey consumption and preference among different developmental stages of *R. fuscipes* for the three prey species were performed using one-way ANOVA, followed by Duncan’s multiple range test (*p* < 0.05).

To determine the type of functional response, cubic logistic regression analysis was conducted to examine the relationship between the proportion of prey consumed and initial prey density as follows:

(1) $$\frac{N_{e}}{N_{0}}=\frac{\exp(P_{0}+P_{1}N_{0}+P_{2}N_{0}^{2}+P_{3}N_{0}^{3})}{1+\exp(P_{0}+P_{1}N_{0}+P_{2}N_{0}^{2}+P_{3}N_{0}^{3})}$$

where *N_e_* represents the number of prey consumed, and *N_0_* denotes the prey density. The parameters *P_0_*, *P*_1_, *P_2_*, and *P_3_* correspond to the intercept, linear, quadratic, and cubic coefficients, respectively [45]. A significant negative linear coefficient (*P*_1_ < 0) indicates a Type II functional response. Conversely, when the linear coefficient is significantly positive (*P*_1_ > 0) and the quadratic coefficient is negative (*P*_2_ < 0), the predator exhibits a Type III functional response [45]. The logistic regression analysis confirmed that the data for each case followed a Type II functional response. Therefore, the random predator equation (Equation (2)) was applied to model the relationship between the number of prey consumed (*Ne*) and the initial prey density (*N_0_*) [56]:

(2) $$N_{e}=N_{0}[1-\exp\left(aT_{h}N_{e}-aT\right)]$$

where *Ne* represents the number of prey consumed, *N_0_* is the initial prey density, *a* denotes the attack rate, *T* is the total searching time (one day), and *T_h_* is the handling time (in days). The ratio *T/T_h_* is used to calculate the theoretical maximum prey consumption, while the functional response rate (FRR = *a/T_h)_* [42] provides an estimate of the predation efficiency of *R. fuscipes*. To compare differences in the attack rate and handling time across the developmental stages of *R. fuscipes* in response to the three prey species, the extra sum-of-squares *F*-test was applied. Parameter estimates were obtained using the non-linear least squares regression procedure.

To determine the effect of predator densities on the prey consumption of *R. fuscipes*, the parameters for intraspecific competition were estimated using non-linear regression analysis by fitting the model described in Equation (3) [57]:

(3) $$E=QP^{-m}$$

In this model, *E* (where $E=N_{e}/\left(N_{0}P\right))$ represents the predation efficency, *p* denotes predator density, *Q* is the search constant, and *m* is the mutual interference constant. A chi-square goodness-of-fit test was performed to evaluate whether the intraspecific competition model appropriately fits the data.

Prey preference was evaluated using Ivlev’s selection index [58], and the selectivity index *C*_i_ was calculated according to Equation (4):

(4) $$C_{i}=\frac{Q_{i}-F_{i}}{Q_{i}+F_{i}}$$

In this equation, *C*_i_ represents the predator’s preference for prey type *i*, *F_i_* refers to the proportion of prey type *i* in the experimental system, and *Q_i_* denotes the proportion of prey type *i* consumed by the predator. A *Ci* value between 0 and 1 indicates a positive preference, a *Ci* value between −1 and 0 indicates a negative preference, and *Ci* = 0 suggests no preference.

All data were checked for normality and homoscedasticity. Statistical analyses were performed using Prism 9.0 (GraphPad), while regression analyses were conducted using SigmaPlot (version 14.0, Systat Software, Inc., San Jose, CA, USA).

## 3. Results

### 3.1. Prey Consumption

**Figure 1:**
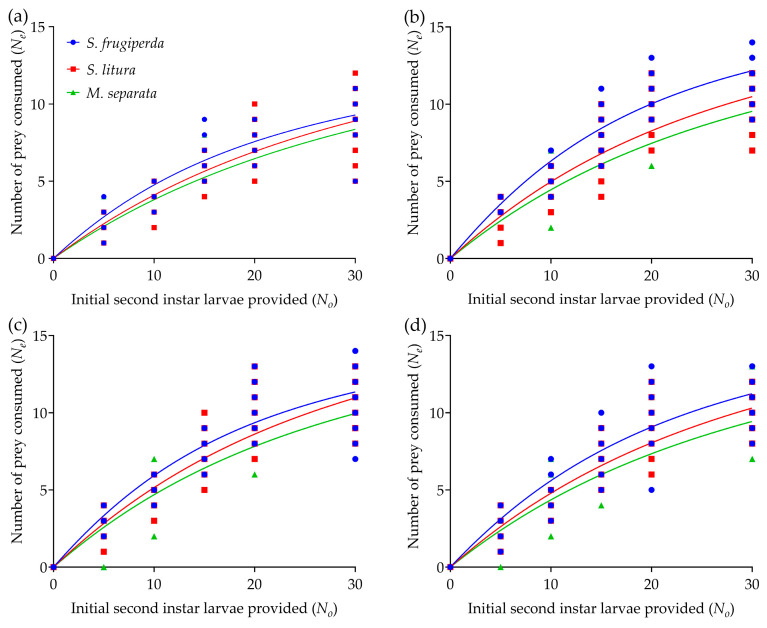
Predation functional responses of the fourth-instar nymph (**a**), fifth-instar nymph (**b**), female adult (**c**), and male adult (**d**) of *R. fuscipes* to the second-instar larvae of *S. frugiperda*, *S. litura*, and *M. separata* at different prey densities. The data points represent the number of prey consumed by *R. fuscipes* over 24 h. The curves represent the predicted values based on Rogers’ random predator equation.

The nymphs and adults of *R. fuscipes* had high consumption of the second-instar larvae of *S. frugiperda*, *S. litura,* and *M. separata*, respectively. In all experimental treatments, the number of prey consumed by *R. fuscipes* over a 24 h period increased significantly with prey density and then plateaued once the prey density reached an upper asymptote (Figure 1). Regardless of prey type, the fourth-instar nymphs of *R. fuscipes* showed the lowest mean daily prey consumption (i.e., consumed 8~9.5 armyworms per day) at the highest prey densities, with no significant differences observed among the fifth-instar nymphs, female and male adults of the predator (Table 1). For all life stages of *R. fuscipes*, predation was highest on *S. frugiperda*, followed by *S. litura*, and lowest on *M. separata* (Table 1). Notably, both the fifth-instar nymphs and male adults of *R. fuscipes* consumed significantly more *S. frugiperda* than *S. litura* and *M. separata*.

### 3.2. Functional Response

The maximum likelihood estimate of the linear parameter *P*_1_ was negative for all life stages of *R. fuscipes* (i.e., *P*_1_ < 0) (Table S1), indicating a Type II functional response of each developmental stage of the predator to each prey species, as revealed by the logistic model analysis (Figure 1). The functional response data for *R. fuscipes* feeding on *S. frugiperda*, *S. litura,* or *M. separata* over a 24 h period closely matched the random predation equation, further supporting the conclusion of a Type II response across all life stages of *R. fuscipes* (Table 2).

With the development of the predator, the attack rate coefficient (*a*) of *R. fuscipes* increases, while the handling time (*T_h_*) decreases (Table 2). For each prey species, both adults and fifth-instar nymphs of *R. fuscipes* generally showed a higher attack rate but shorter handling time compared to earlier developmental stages (Table 2). The functional response parameters of the predator were also influenced by the prey species. For each developmental stage of *R. fuscipes*, the highest attack rate was observed in predators feeding on *S. frugiperda,* with no significant differences observed between predators feeding on the *S. litura* and *M. separata* (*F*= 60.829, *df* = 57, *p* < 0.0001). Although the female adults of *R. fuscipes* feeding on *S. litura* displayed the shortest handling time, there were no significant differences among the three prey species (Table 2). Based on the theoretical maximum consumption parameter (*T*/*T_h_*), the consumption rate by *R. fuscipes* increased with developmental stage, with female adults showing greater predation on *S. litura* than on other prey species. The predatory efficiency of *R. fuscipes* can be more objectively and accurately reflected by the functional response ratio (FRR *= a*/*T_h_*) [19,59], where a higher FRR value indicates greater predation efficiency. Among the various life stages, *R. fuscipes* exhibited the highest predation efficiency on *S. frugiperda* and the lowest on *M. separata.* Fifth-instar nymphs and adults generally had higher FRR values than fourth-instar nymphs. The highest predation efficiency was observed in the fifth-instar nymph of *R. fuscipes* feeding on *S. frugiperda* (FRR = 28.0718), followed by the female adults (FRR = 23.7099) and male adults (FRR = 21.9453) (Table 2).

### 3.3. Intraspecific Interference Competition

**Figure 2:**
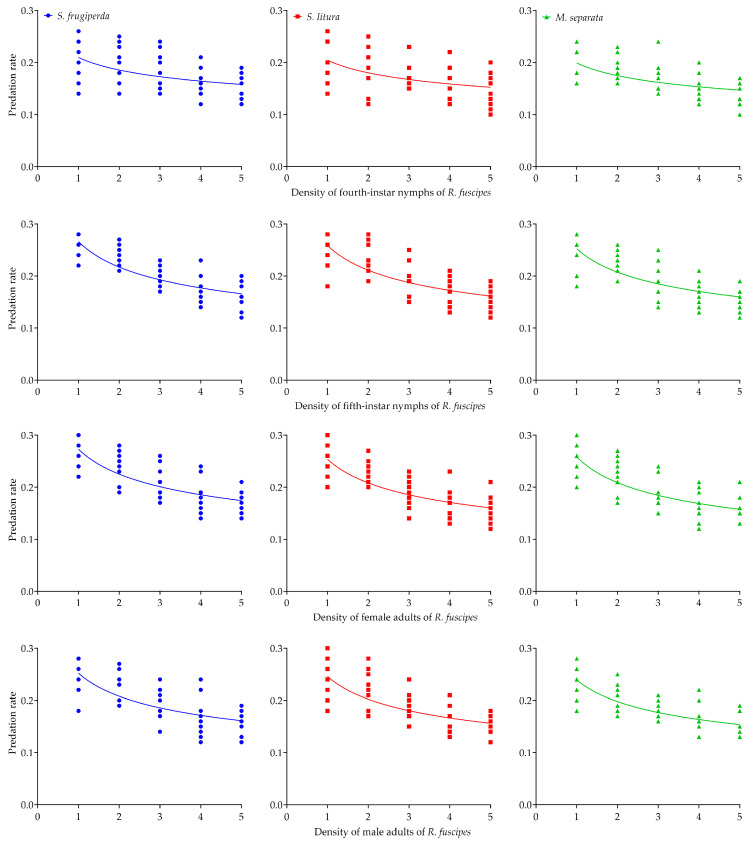
The intraspecific interference competition of the fourth-instar and fifth-instar nymphs as well as the female adults and male adults of *R. fuscipes* preying on the second-instar larvae of *S. frugiperda*, *S. litura,* and *M. separata*, respectively.

All models converged on non-zero estimates for the predator interference parameter. When the prey-to-predator ratio was kept at 50, an increase in both predator and prey numbers led to a gradual rise in the total consumption of the three prey species by *R. fuscipes* in the cages. Across all conditions, the average daily consumption of each prey species by *R. fuscipes* at different life stages significantly decreased as predator density increased (Table 3, Figure 2). For all predator–prey density combinations tested, the intraspecific interference competition model fit the empirical data well, as indicated by the asymptotic 95% confidence intervals excluding zero and relatively high R-squared values (Table 4).

The intraspecific competition model effectively captured the predation rates at varying densities of *R. fuscipes*, regardless of prey species (Table 4). The asymptotic 95% confidence intervals, which did not include zero, were evident in the quest search constant (*Q*) and mutual interference constant (*m*) for all life stages of *R. fuscipes*. In nearly every case, *R. fuscipes* showed the highest predation rate on *S. frugiperda* and the lowest on *M. separata*. The exception was observed in the female adults of *R. fuscipes*, where the predation rate on *M. separata* exceeded that on *S. litura*. As *R. fuscipes* developed, intraspecific interference competition among the predators progressively intensified (Table 4, Figure 2).

### 3.4. Prey Preference

**Figure 3:**
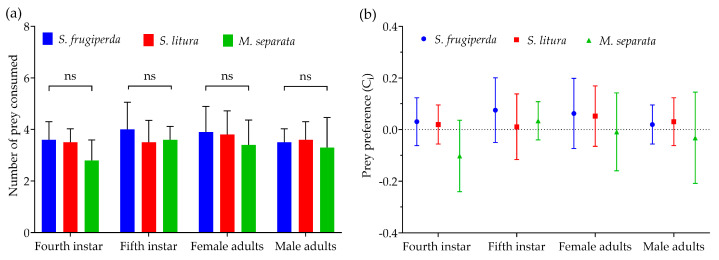
The mean consumption rate (**a**) and prey preference (**b**) of the fourth-instar and fifth-instar nymphs as well as the female adults and male adults of *R. fuscipes* consuming second-instar larvae of *S. frugiperda*, *S. litura*, and *M. separata*, respectively.

When all three prey species were offered simultaneously, *R. fuscipes* showed higher predation on *S. frugiperda* than on *S. litura* or *M. separata* at every life stage, although the differences were not statistically significant (Figure 3a). All life stages of *R. fuscipes* demonstrated a positive preference for the second-instar larvae of *S. frugiperda* and *S. litura*, while showing a negative preference for *M. separata*, except for the fifth-instar nymphs of the predator (Figure 3b).

## 4. Discussion

Our results suggest that the reduviid predator *R. fuscipes* has potential as an effective biocontrol agent against the major agricultural pests *S. frugiperda*, *S. litura,* and *M. separata*. This is primarily due to the greater predatory efficiency exhibited by older nymphs and adults of *R. fuscipes*, which were more effective at preying on the second-instar larvae of each prey species. Notably, *R. fuscipes* nymphs and adults, particularly the fifth-instar nymphs, demonstrated a higher consumption rate and preference for *S. frugiperda* over *S. litura* and *M. separata*. A similar pattern was observed in the fifth-instar nymphs of the closely related reduviid predator *R. marginatus*, which also consumed more *S. frugiperda* larvae than other prey life stages [33]. However, the female adults of *R. fuscipes* consistently exhibited the highest predation rates on *S. litura* and *M. separata* larvae compared to other developmental stages. This finding is in line with the work of George and Seenivasagan, who found that the female adults of *R. marginatus* had the highest predation rates on *S. litura* [60]. These results indicate that both the adult and fifth-instar nymph stages of *R. fuscipes* are particularly suitable for potential use in biological control programs.

Type II functional responses are commonly observed in insect predators [19,61,62], including predatory stinkbugs such as *O. sauteri* (Poppius) [63], *A. chinensis* [27], *P. lewisi* [32], *Dicyphus bolivari* (Lindberg), and *Dicyphus errans* (Wolff) [43]. In our study, *R. fuscipes* displayed Type II functional responses at the adult and fifth- and fourth-instar nymphal stages when preying on the larvae of *S. frugiperda*, *S. litura,* and *M. separata*. Previous studies have also reported Type II functional responses in *R. fuscipes* when feeding on other pests, such as the cotton mealybug *P. solenopsis* [36], the red cotton bug *D. koenigii* [36], the Russian wheat aphid *Diuraphis noxia* (Mordvilko) [47], the cucumber leaf folder *Diaphania indicus* (Saunders) [64], and the teak skeletonizer *Eutectona machaeralis* (Walker) [65]. In contrast, some predatory species, such as the minute pirate bug *Anthocoris minki* (Dohrn) preying on *Psyllopsis repens* [48] and *Nabis* sp. preying on both the leafhopper species *Agallia constricta* and *Ceratagallia agricola* [66], have shown a Type III functional response. In these cases, prey consumption remains low at low prey densities but increases at higher densities, a characteristic of Type III functional responses. Type II functional responses may lead to instability in predator–prey dynamics, as high predation rates at low prey densities can cause prey population collapse. Conversely, Type III functional responses are considered more stabilizing, as they allow for prey refuge at low densities or alternative prey switching, while increasing foraging efficiency at high prey densities [62,67]. Thus, predators exhibiting Type II functional response may be more effective in controlling pest populations at lower prey densities, making them ideal candidates for augmentative biological control strategies, especially when predator–prey ratios are high.

However, under complex field conditions, the functional response of predators exhibiting Type II behaviors could shift to Type III due to factors such as temperature [68], predator developmental stage [67], starvation levels [69], prey switching [70], host plants [47], space availability [71], and foraging area size [61]. A precise and reliable identification of functional response types is critical for understanding predator–prey dynamics and informing the optimal use of biocontrol agents. Experimental settings with limited prey availability may result in estimates of functional responses that represent theoretical maximum interactions under those specific conditions. Kalinkat et al. emphasized that the importance of considering appropriate prey density ranges, habitat structure, and biologically relevant designs when conducting functional response experiments [72]. Therefore, it is crucial to conduct further studies on the functional response and predatory efficiency of *R. fuscipes* under semi-field or field conditions targeting a range of pests across various crops. Such studies will provide more robust insights into the predator’s potential as a biocontrol agent and inform its practical application in integrated pest management systems.

The key indicators of predatory functional responses are the attack rate and handling time, which reflect the theoretical efficiency of predators in capturing, killing, and consuming prey [73]. Our results clearly demonstrate the significant influence of both prey species and predator developmental stages on these two key parameters (Table 2). At the same developmental stage, *R. fuscipes* exhibits distinct species-specific differences in attack rates and prey handling times when preying on three noctuid pests with similar host ranges and body sizes. These variations underscore the complexity of foraging strategies employed by *R. fuscipes*. Moreover, as predators mature, their body size, strength, and predatory skills improve, potentially leading to increased attack rates and enhanced handling efficiency [70]. For instance, when preying on *S. litura* and *M. separata*, both the attack rates and handling efficiency of *R. fuscipes* increased progressively with its development. Interestingly, when preying on *S. frugiperda*, the fifth-instar nymphs of *R. fuscipes* exhibited a significantly higher attack rate than adults, while prey handling efficiency did not differ substantially between the two stages. This finding is similar to the observations of Sahayaraj et al., where the fifth-instar nymphs of *Rhynocoris longifrons* (Stål) had higher attack rates than adults when preying on *Aphis gossypii* (Glover) [74]. However, in *Rhynocoris kumarii* Ambrose, the attack rate of fifth-instar nymphs towards the prey *P. solenopsis* Tinsley was higher than that of male adults but lower than that of female adults, with significant differences in prey handling efficiency [75]. Additional factors, such as temperature [76,77], host plant species [78], search area [77], hunger level [67,79] and insecticide exposure [80,81], also significantly influence attack rates and handling times. However, relying solely on attack rates and handling times may lead to contradictory predictions regarding predatory efficiency. To address this, a new metric, the parameter FRR (*a/T_h_*) has been proposed [19]. Our results indicate that the FRR (*a/T_h_*) of *R. fuscipes* is also influenced by both prey species and predator developmental stage. When *S. litura* was the prey, *R. fuscipes* reached maximum predatory efficiency at the adult stage. These results suggest that predators at different developmental stages may encounter different ecological challenges and predation pressures, leading to hierarchical differences in their functional responses.

Intraspecific competition occurs when predators of the same species compete for resources, such as food or territory, and can significantly impact behavior, population dynamics, and ecosystem functioning [82]. Our results indicate that when the prey-to-predator ratio was maintained at 50, the average daily consumption of the three noctuid pests by *R. fuscipes* decreased as the predator density increased (Table 3 and Table 4). This decline in predation efficiency is likely due to intensified spatial competition and resource contention at higher predator densities. Additionally, the interaction between prey species and predator developmental stage appears to modulate intraspecific interference. Regardless of prey type, the fifth-instar nymphs and adults of *R. fuscipes* experienced significantly stronger intraspecific interference than the fourth-instar nymphs. This was particularly evident when *S. frugiperda* and *M. separata* were the prey, with the highest levels of intraspecific interference occurring at the fifth-instar nymph and adult stages of *R. fuscipes*, likely due to maximum prey consumption at these stages, which intensified competition for resources (Table 1). Interestingly, although *R. fuscipes* adults consumed the most *S. litura*, intraspecific interference was more pronounced during the fifth-instar nymph stage. This suggests that other factors, such as the hunger level of predators, may also play a significant role in shaping interference dynamics [83]. Studies have shown that the hunger level of predators can influence intraspecific competition; once predators reach satiety, their activity levels increase, and they shift from foraging to mating behaviors, which can exacerbate interference, especially among same-sex adults [81,82,83]. This may partially explain the stronger interference effects observed in the adult stage compared to other developmental stages of *R. fuscipes*. Understanding intraspecific competition is crucial for optimizing predator population management and enhancing pest control efficiency, allowing for reduced interference while maintaining high predation performance. However, the optimal release ratio of natural enemies remains to be validated through field trials.

In field conditions, the presence of alternative prey (non-target pest) may alter the predation efficiency and functional response of generalist predators to target pests [84,85]. Given the variety of available prey, understanding the predator’s feeding preferences is critical for enhancing its application in biological control programs [19]. In our study, the generalist predator *R. fuscipes* at different developmental stages showed a preference for consuming *S. frugiperda* and *S. litura* over *M. separata* in a multi-prey system. This aligns with findings by Nagarajan et. al., who used a Y-shaped olfactometer to analyze the prey preferences of adult *R. fuscipes*. They reported that *R. fuscipes* exhibited the highest preference for *S. litura* compared to other prey species such as *Helicoverpa armigera* Hubner, *Achaea janata* Linnaeus, and *D. cingulatus* and *Mylabris indica* Thunberg [86]. However, the fifth-instar nymphs of *R. fuscipes* showed a positive selectivity towards *M. separata*. The feeding preferences of predators may be influenced by factors such as prey size, nutritional quality, behavioral traits, and chemical cues [85,87]. For example, *R. fuscipes* prefers feeding on soft-bodied prey such as termites (*Odontotermes obesus* Rambur) over more chitinized prey such as grasshoppers (*Oxya* sp.) [88]. Similarly, when offered a choice between *D. cingulatus*, *S. litura,* and castor pest *A. janata*, *R. fuscipes* responded most strongly to a chloroform–methanol extract of *A. janata*, with the least response to *S. litura* extract [89]. Given that the three noctuids (*S. frugiperda*, *S. litura,* and *M. separata*) used in this study share similar host plant ranges [8,9,10], we hypothesize that chemical pheromones released by *S. frugiperda* or *S. litura* may attract *R. fuscipes* to these prey. In natural field settings, these three armyworms often occur simultaneously, highlighting the need for further studies on the predatory behavior and consumption rates of *R. fuscipes* in complex multi-prey environments.

It is important to note that *S. frugiperda*, *S. litura,* and *M. separata* enter a highly destructive feeding phase following the third-instar, which significantly exacerbates their damage to crops [90,91,92]. Effective population control prior to the end of the third-instar can substantially reduce crop loss and economic losses. This study focuses primarily on evaluating the biological control efficacy of various developmental stages of *R. fuscipes* against the second-instar larvae of these pest species. Since eggs and first-instar larvae are more vulnerable to natural enemies and third-instar larvae represent a critical stage in the pest’s voracious feeding cycle, further exploration of *R. fuscipes*’ predation capabilities against their earlier and later pest stages is warranted. This will enhance our understanding of its functional response and provide a basis for developing more targeted and efficient pest management strategies, ultimately improving the application of *R. fuscipes* in integrated pest management.

## 5. Conclusions

In conclusion, we provide evidence that *R. fuscipes* is a promising biological control agent for controlling early larval stages of *S. frugiperda*, *S. litura,* and *M. separata*. The most effective predatory stages are the fifth-instar nymphs and adults, which have higher foraging efficiency compared to earlier developmental stages. *R. fuscipes* showed a Type II functional response to all prey species, suggesting that it is likely to be more effective in suppressing noctuid populations at lower prey densities. Intraspecific competition among *R. fuscipes* individuals intensifies with both predator development and increasing population density. Notably, *R. fuscipes* appears to be more efficient in controlling *S. frugiperda* than the other two noctuid pests, as evidenced by its superior predation capacity and prey preference. Considering factors such as predatory effectiveness and intraspecific interference, our findings suggest that releasing fourth-instar nymphs of *R. fuscipes* during periods of low noctuid larval density would optimize the efficacy of biological control. These results contribute valuable insights into predator–prey dynamics, predator–predator interactions, and the foraging behavior of *R. fuscipes*. However, further studies are needed to assess its utility in managing armyworms across various crops and in complex agroecosystems. Future research should focus on evaluating the influence of prey stage, prey switching, predator–prey ratio, and environmental factors such as temperature on the predation efficiency of *R. fuscipes* under field conditions. This will be essential for developing a comprehensive and effective integrated pest management strategy.

## Figures and Tables

**Table 1:** Prey consumption (±SE) per day for nymphs and adults of *R. fuscipes* of the second-instar larvae of *S. frugiperda*, *S. litura,* and *M. separata* at the highest prey density, respectively.

*R. fuscipes*	*S. frugiperda*	*S. litura*	*M. separata*	*F*, *p*
Fourth-instar nymphs	9.05 ± 0.29 Ab	8.65 ± 0.37 Ab	8.15 ± 0.31 Ab	*F*_(2, 57)_ = 1.839, *p* = 0.168
Fifth-instar nymphs	11.75 ± 0.37 Aa	10.05 ± 0.32 Ba	9.15 ± 0.33 Ba	*F*_(2, 57)_ = 14.272, *p* < 0.0001
Female adults	10.90 ± 0.40 Aa	10.45 ± 0.36 ABa	9.50 ± 0.24 Ba	*F*_(2, 57)_ = 4.228, *p* < 0.05
Male adults	10.85 ± 0.32 Aa	9.85 ± 0.32 Ba	9.05 ± 0.30 Ba	*F*_(2, 57)_ = 7.854, *p* < 0.01
*F*, *p*	*F*_(3, 76)_ = 10.268, *p* < 0.0001	*F*_(3, 76)_ = 4.877, *p* < 0.05	*F*_(3, 76)_ = 3.513, *p* = 0.019	

Each value represents the mean ± SE (*n* = 20). Means within the same row followed by different capital letters are significantly different among the different species of prey consumed by the same developmental stage of *R. fuscipes* (one-way ANOVA followed by Duncan’s multiple comparisons, *p* < 0.05). Means within the same column followed by different lowercase letters are significantly different for different developmental stages of *R. fuscipes* consuming the same prey species (one-way ANOVA followed by Duncan’s multiple comparisons, *p* < 0.05).

**Table 2:** Parameter estimates from the Holling II functional response of different life stages of *R. fuscipes* preying on the second-instar larvae of *S. frugiperda*, *S. litura*, and *M. separata*, respectively.

*R. fuscipes*	Prey Species	Attack Rate ± SE (*a*)	Handling Time ± SE (*T_h_*)	Maximum Consumption (*T/T_h_*)	FRR (*a/T_h_*)	*R* ^2^
Fourth-instar nympha	*S. frugiperda*	0.9390 ± 0.0364 a	0.0650 ± 0.0030 a	15.3752	14.3804	0.8875
	*S. litura*	0.6820 ± 0.0503 b	0.0533 ± 0.0038 a	18.5117	12.5546	0.8750
	*M. separata*	0.6125 ± 0.0389 b	0.0547 ± 0.0052 a	18.1422	10.9851	0.8786
*F*, *p*		*F*_(2, 57)_ = 12.394, *p* < 0.05	*F*_(2, 57)_ = 1.836, *p* = 0.214			
Fifth-instar nympha	*S. frugiperda*	1.5205 ± 0.0544 a	0.0540 ± 0.0023 a	18.5048	28.0718	0.9217
	*S. litura*	0.9272 ± 0.0458 b	0.0507 ± 0.0025 a	19.6580	18.1168	0.8860
	*M. separata*	0.7748 ± 0.0263 b	0.0531 ± 0.0028 a	18.9036	14.5898	0.8749
*F*, *p*		*F*_(2, 57)_ = 60.829, *p* < 0.0001	*F*_(2, 57)_ = 0.337, *p* = 0.723			
Female adult	*S. frugiperda*	1.3913 ± 0.1010 a	0.0575 ± 0.0064 a	17.4338	23.7099	0.9087
	*S. litura*	0.9780 ± 0.0446 b	0.0488 ± 0.0052 a	20.7512	19.9875	0.8866
	*M. separata*	0.8489 ± 0.0486 b	0.0522 ± 0.0038 a	19.1534	16.0563	0.8756
*F*, *p*		*F*_(2, 57)_ = 12.422, *p* < 0.05	*F*_(2, 57)_ = 0.533, *p* = 0.604			
Male adult	*S. frugiperda*	1.1778 ± 0.0619 a	0.0534 ± 0.0021 a	18.7406	21.9453	0.8969
	*S. litura*	0.8578 ± 0.0254 b	0.0499 ± 0.0049 a	20.3666	17.3381	0.8929
	*M. separata*	0.7555 ± 0.0521 b	0.0524 ± 0.0078 a	19.2864	14.2469	0.8760
*F*, *p*		*F*_(2, 57)_ = 15.185, *p* = 0.001	*F*_(2, 57)_ = 0.085, *p* = 0.919			

Each value represents the mean ± SE (*n* = 20). For each developmental stage of *R. fuscipes*, means within the same column followed by different lowercase letters indicate significant differences among prey species (one-way ANOVA followed by Duncan’s multiple comparisons, *p* < 0.05). *R*^2^ is the coefficient of determination obtained by fitting Holling’s disk equation.

**Table 3:** Average daily predation rate by different life stages of *R. fuscipes* on second-instar larvae of *S. frugiperda*, *S. litura* and *M. separata* at different predator densities.

*R. fuscipes*	Prey Species	Prey-Predator Ratio				
		50:1	100:2	150:3	200:4	250:5
Fourth-instar nymphs	*S. frugiperda*	10.10 ± 0.61 a	9.75 ± 0.56 a	9.27 ± 0.54 ab	8.00 ± 0.39 bc	7.38 ± 0.39 c
	*S. litura*	9.90 ± 0.57 a	9.45 ± 0.63 a	8.77 ± 0.44 ab	7.70 ± 0.48 b	7.26 ± 0.51 b
	*M. separata*	9.60 ± 0.45 a	9.30 ± 0.35 a	8.57 ± 0.43 a	7.20 ± 0.39 b	7.10 ± 0.35 b
Fifth-instar nymphs	*S. frugiperda*	12.70 ± 0.35 a	12.07 ± 0.32 a	9.82 ± 0.38 b	8.24 ± 0.47 c	7.85 ± 0.40 c
	*S. litura*	12.40 ± 0.47 a	11.60 ± 0.43 a	9.67 ± 0.45 b	8.08 ± 0.44 c	7.60 ± 0.36 c
	*M. separata*	12.10 ± 0.46 a	11.40 ± 0.30 a	9.50 ± 0.51 b	7.95 ± 0.38 c	7.60 ± 0.31 c
Female adults	*S. frugiperda*	13.10 ± 0.48 a	12.35 ± 0.49 a	10.33 ± 0.49 b	8.90 ± 0.50 c	8.08 ± 0.35 c
	*S. litura*	12.20 ± 0.53 a	11.45 ± 0.32 a	9.40 ± 0.40 b	8.08 ± 0.45 c	7.58 ± 0.41 c
	*M. separata*	12.60 ± 0.45 a	11.20 ± 0.49 b	9.17 ± 0.44 c	7.88 ± 0.47 c	7.78 ± 0.39 c
Male adults	*S. frugiperda*	12.10 ± 0.50 a	11.40 ± 0.41 a	9.57 ± 0.45 b	8.20 ± 0.55 bc	7.56 ± 0.38 c
	*S. litura*	11.90 ± 0.64 a	10.95 ± 0.57 a	9.17 ± 0.41 b	7.90 ± 0.48 bc	7.52 ± 0.31 c
	*M. separata*	11.80 ± 0.46 a	10.20 ±0.39 b	9.00 ± 0.26 c	7.85 ± 0.44 cd	7.50 ± 0.39 d

Each value represents the mean ± SE (*n* = 20). Means within the same row followed by different lowercase letters indicate significant differences (*p* < 0.05) in the predation rate by *R. fuscipes* at different predator densities, as determined by one-way ANOVA followed by Duncan’s multiple comparisons.

**Table 4:** Parameters estimated from the intraspecific competition interference equation of different life stages of *R. fuscipes* preying on the second-instar larvae of *S. frugiperda*, *S. litura,* and *M. separata*, respectively.

*R. fuscipes*	Prey Species	Maximum Predation (*Q*)	95% Confidence Interval (CI)	Interference Coefficient (*m*)	95% Confidence Interval (CI)	*R* ^2^
Fourth-instar nympha	*S. frugiperda*	0.2097	0.1906–0.2292	0.1765	0.08671–0.2650	0.2410
	*S. litura*	0.2045	0.1846–0.2247	0.1828	0.08666–0.2776	0.2290
	*M. separata*	0.1992	0.1837–0.2149	0.1887	0.1118–0.2647	0.3298
Fifth-instar nympha	*S. frugiperda*	0.2661	0.2497–0.2826	0.2939	0.2302–0.3575	0.6305
	*S. litura*	0.2586	0.2410–0.2763	0.2930	0.2228–0.3630	0.5831
	*M. separata*	0.2523	0.2357–0.2697	0.2837	0.2158–0.3513	0.5836
Female adults	*S. frugiperda*	0.2730	0.2543–0.2919	0.2790	0.2084–0.3492	0.5570
	*S. litura*	0.2538	0.2364–0.2713	0.2854	0.2147–0.3559	0.5669
	*M. separata*	0.2583	0.2408–0.2760	0.3075	0.2364–0.3784	0.5999
Male adults	*S. frugiperda*	0.2521	0.2335–0.2709	0.2779	0.2019–0.3536	0.5183
	*S. litura*	0.2459	0.2267–0.2653	0.2815	0.2008–0.3619	0.4946
	*M. separata*	0..2392	0.2243–0.2542	0.2744	0.2101–0.3385	0.5940

## Data Availability

The data presented in this study are available on request from the corresponding author.

## Supplementary Materials

The following supporting information can be downloaded at https://www.mdpi.com/article/10.3390/insects16020224/s1, Table S1: Logistic models describing the proportion of second-instar larvae of *S. frugiperda*, *S. litura,* and *M. separata* consumed by different developmental stages of *R. fuscipes* as a function of initial prey density.
