# Supplementary material for: Biological Control Potential of the Reduviid Predator Rhynocoris fuscipes (Fabricius) in Managing Noctuid Pests: Insights Into Predation and Prey Preference
Source: Insects. 2025 Feb 18;16(2):224. doi: 10.3390/insects16020224 (PMC11856166; doi:10.3390/insects16020224)
Supplement: Supplementary file 1 [file insects-16-00224-s001.zip › insects-3419977-supplementary.pdf]

insects

MDPI

Table S1. Logistic models describing the proportion of second-instar larvae of *S. frugiperda*, *S. litura* and *M. separata* consumed by different developmental stages of *R. fuscipes* as a function of initial predy density.

R. fuscipes	Prey Species	Type	Parameters	Estimate	SE	*t*	*P*
Fourth instar nympha	*S. frugiperda*	II	P_0	1.2543	0.5539	2.2643	0.0254
			P_1	−0.2716	0.1072	−2.5341	0.0126
			P_2	0.0151	0.0063	2.4100	0.0175
			P_3	−0.0003	0.0001	−2.5231	0.013
	S. litura	II	P_0	0.8537	0.5618	1.5197	0.1313
			P_1	−0.2705	0.1097	−2.4668	0.0151
			P_2	0.0164	0.0065	2.5395	0.0124
			P_3	−0.0003	0.0001	−2.7167	0.0076
	M. separata	II	P_0	0.1052	0.5488	0.1917	0.8483
			P_1	−0.1394	0.1070	−1.3035	0.195
			P_2	0.0086	0.0063	1.3593	0.1767
			P_3	−0.0002	0.0001	−1.5297	0.1288
Fifth instar nympha	*S. frugiperda*	II	P_0	1.7171	0.6395	2.6850	0.0083
			P_1	−0.2530	0.1209	−2.0936	0.0385
			P_2	0.0144	0.0070	2.0647	0.0412
			P_3	−0.0003	0.0001	−2.2816	0.0243
	S. litura	II	P_0	0.4732	0.6087	0.7774	0.4385
			P_1	−0.1478	0.1176	−1.2561	0.2116
			P_2	0.0102	0.0069	1.4759	0.1427

			P_3	−0.0002	0.0001	−1.7875	0.0765
	M. separata	II	P_0	0.1040	0.6038	0.1723	0.8635
			P_1	−0.1117	0.1168	−0.9564	0.3409
			P_2	0.0080	0.0068	1.1673	0.2455
			P_3	−0.0002	0.0001	−1.4587	0.1473
Female adult	*S. frugiperda*	II	P_0	1.4272	0.6138	2.3252	0.0218
			P_1	−0.2338	0.1170	−1.9985	0.048
			P_2	0.0136	0.0068	2.0091	0.0469
			P_3	−0.0003	0.0001	−2.2495	0.0264
	S. litura	II	P_0	0.3782	0.6232	0.6069	0.5451
			P_1	−0.1309	0.1203	−1.0880	0.2789
			P_2	0.0098	0.0070	1.4000	0.1642
			P_3	−0.0002	0.0001	−1.7713	0.0791
	M. separata	II	P_0	0.1257	0.6145	0.2045	0.8383
			P_1	−0.1066	0.1187	−0.8976	0.3713
			P_2	0.0080	0.0069	1.1590	0.2488
			P_3	−0.0002	0.0001	−1.4919	0.1384
Male adult	*S. frugiperda*	II	P_0	1.1591	0.6417	1.8064	0.0734
			P_1	−0.2098	0.1231	−1.7050	0.0909
			P_2	0.0125	0.0072	1.7465	0.0834
			P_3	−0.0002	0.0001	−1.9647	0.0518
	S. litura	II	P_0	0.0902	0.5758	0.1567	0.8758
			P_1	−0.0958	0.1112	−0.8615	0.3908
			P_2	0.0076	0.0065	1.1673	0.2455

		P_3	−0.0002	0.0001	−1.5364	0.1272
M. separata	II	P_0	0.1144	0.5950	0.1924	0.8478
		P_1	−0.1251	0.1151	−1.0862	0.2796
		P_2	0.0089	0.0067	1.3223	0.1887
		P_3	−0.0002	0.0001	−1.6239	0.1071
